# Supplementary material for: Clustering of the structures by using “snakes-&-dragons” approach, or correlation matrix as a signal
Source: PLoS One. 2019 Oct 10;14(10):e0223267. doi: 10.1371/journal.pone.0223267 (PMC6786638; doi:10.1371/journal.pone.0223267)
Supplement: S1 Appendix — (DOCX) [file pone.0223267.s001.docx]

Supplemental Material. Scaling and Weighting Variables in Constructing Dragon Vectors

One of the important choices in clustering any vectors, not necessarily snake or dragon vectors, is whether to standardize or not to standardize the variables. As described in [S1], the problem with unstandardized data is the inconsistency between cluster solutions when the scale of variables is changed, which is a strong argument in favor of standardization. The common form of conversion of variables to standard scores (or z-scores) entails subtracting the mean and dividing by the standard deviation for each variable. However, standardization defined in this way is not always suitable. For example, when defining disease subtypes, it is important to know whether variability is caused by natural biological variability of the patients or by the differences in disease subtypes. Subtracting the overall mean and dividing by the overall standard deviation would mask the subtype differences. The solution to this problem is standardization by the mean and standard deviation of a control subjects group, who do not have the disease of interest. In doing this, one could separate natural biological variability captured by the standard deviations of controls from the variability reflective of the differences between disease subtypes or different diseases [S2].

Another problem is that clustering results can be skewed if based on multiple variables reflecting redundant information. One possible solution is weighting of the variables so that the highest weight is attributed to the least correlated variables and the lowest weight attributed to the most correlated variables [S3]. A more systematic approach is to create orthogonal (uncorrelated) variables by performing principal components analysis prior to clustering. The next choice is whether to use in clustering all principal components (PC) with equal weights (“unweighted PC”), select only several PCs that explain a high percentage of variance (“truncated PC”), or weight each PC by its percentage of variance explained (“weighted PC”). The latter option seems advantageous, since it accounts for explained variance and does not rely on an arbitrarily chosen threshold. We believe that there is no universal solution to these problems, which are not dragon vector-specific, but are relevant to any clustering decision.

Below, we illustrate how these choices can affect clustering of dragon vectors by using data on microbiome dynamics of 52 healthy students [S4]. In the main body of the paper, we calculated the correlations across OTU counts observed at seven time points (weeks) at four body sites (gut, tongue, palm, and forehead) to explore the temporal changes in each subject’s microbiome. We created N x N=7x7 correlation matrices for each person and each body site to represent the similarities between the observed seven weeks in terms of the microbiome composition. Then, we created snake vectors for each of the matrices and combined them into dragon vectors representing the microbiome dynamics of four body sites for each subject.

We made explicit decisions on which variables to include. First, we decided to use only the information about microbiomes and not about the hosts (subjects). Second, we limited ourselves to the overall information on microbiomes reflected by the number of OTUs and Shannon Index, characterizing the diversity of the microbiome at each site, each week, and for each of the subjects. Thus, for each subject, we calculated means and standard deviations of the number of OTUs and of Shannon Index values across seven weeks for each of the four body sites, which resulted in 16 variables representing overall state and variability of the microbiome. Since each of the snake vectors is N(N-1)/2 long, we now have dragon vectors which consist of 100 variables: 16 characterizing overall state and overall variability of the microbiome and 84 characterizing the microbiome time profile. While the values of 84 snake vector variables are within the interval [-1,1], the numbers of OTUs are in the hundreds and thousands. Therefore, we scaled all the variables by calculating z-scores and then performed PCA in order to mitigate redundancy of the variables as discussed above. Next, we explored several approaches: (a) unweighted PC using all 52 principal components with equal weights, (b) truncated PC using only the first 29 PCs that explain 90% of variance, and (c) weighted PC using all 52 PCs and their percentage of variance explained. Clustering was performed using the resampling-based consensus clustering approach of Monti et al [S5] briefly described in the Methods section. The number of clusters was scanned from k=2 to 8, and then three quality of clustering criteria (Calinski [S6], Davies [S7], and Silhouette [S8]) were evaluated. Note that high quality of clustering is indicated by high values of Calinski and Silhouette criteria and low values of Davies criterion. According to all three criteria, the optimal number of clusters was k=2, and the best quality of clustering was observed using the weighted PC approach. Suppl. Table 1 provides the values of the above three criteria for k=2 ,3, … 8 using the weighted PC approach, while Suppl. Table 2 compares the values of the criteria in three weighting approaches, demonstrating the advantage of the weighted PC approach in this particular case.

Supplemental Table 1. Values of quality of clustering criteria for k=2, 3, … 8 in the weighted PC approach

| Number of Clusters, k | Calinski | Davies | Silhouette |
| --- | --- | --- | --- |
| 2 | 49.75 | 0.933 | 0.393 |
| 3 | 41.29 | 1.140 | 0.311 |
| 4 | 35.43 | 1.231 | 0.267 |
| 5 | 29.85 | 1.201 | 0.229 |
| 6 | 27.83 | 1.308 | 0.181 |
| 7 | 25.91 | 1.223 | 0.189 |
| 8 | 23.89 | 1.127 | 0.193 |

Supplemental Table 2. Values of quality of clustering criteria k=2 in three weighting approaches

| Approach | Calinski | Davies | Silhouette |
| --- | --- | --- | --- |
| Unweighted PC | 7.07 | 2.59 | 0.099 |
| Truncated PC | 7.91 | 2.45 | 0.113 |
| Weighted PC | 49.75 | 0.933 | 0.393 |

Although the differences in the criteria values were substantial, the difference in cluster membership was not that dramatic and differed by one subject in comparison of unweighted and truncated PC, and by two subjects in comparison of truncated and weighted PC. Also interestingly, the truncated PC approach performed slightly better than the unweighted PC approach in this particular case. Obviously, this one example cannot and is not intended to prove the universal advantage of the weighted PC approach. The choice of the weighting approach depends on the specific data set. We anticipate that in some situations, especially in cases of Big Data, the truncated PC approach could be preferential since it will be less computationally intense than the weighted PC approach.

Supplemental References

S1. Hair JR, Anderson RE, Tatham RL, Black WC (1998) Multivariate Data Analysis. Prentice- Hall Inc, Upper Saddle River, NJ.

S2. Andreev VP, Gillespie BW, Helfand BT, Merion RM. Misclassification errors in unsupervised classification methods. Comparison based on the simulation of targeted proteomics data. J Proteomics Bioinform. 2016; S14:005.

S3. Andreev VP, Gang L, Yang CC et al. Symptom-based Clustering of Women in the Symptoms of Lower Urinary Tract Dysfunction Research Network (LURN) Observational Cohort Study. J Urol 2018; 200(6) 1323-1331.

S4. Flores GE, Caporaso JG, Henley JB, Rideout JR, Domogala D, Chase J, et al. Temporal variability is a personalized feature of the human microbiome. Genome Biology. 2014; 15:531.

S5. Monti S, Tamayo P, Mesirov J, Golub T. Consensus clustering: A resampling-based method for class discovery and visualization of gene expression microarray data. Machine Learning. 2003; 52:91-118.

S6. Calinski RB, Harabasz J. A dendrite method for cluster analysis. Commun Stat. 1974; 3:1-27.

S7. Davies, DL, Bouldin DW. A Cluster Separation Measure. IEEE Trans Pattern Anal Mach Intell. 1:224–227, 1979.

S8. Rouseeuw PJ. Silhouettes: A graphical aid to the interpretation and validation of cluster analysis. J Comput Appl Math. 1987; 20(1):53-65.
